# A theoretical approach for a new design of an ultrasensitive angular plasmonic chemical sensor using black phosphorus and aluminum oxide architecture

**DOI:** 10.1039/d3ra01984e

**Published:** 2023-05-30

**Authors:** Abdulkarem H. M. Almawgani, Suneet Kumar Awasthi, Ahmed Mehaney, Ghassan Ahmed Ali, Hussein A. Elsayed, Hassan Sayed, Ashour M. Ahmed

**Affiliations:** a Electrical Engineering Department, College of Engineering, Najran University Najran Kingdom of Saudi Arabia; b Department of Physics and Material Science and Engineering, Jaypee Institute of Information Technology Noida 201304 U.P. India; c Physics Department, Faculty of Science, Beni-Suef University Beni-Suef 62512 Egypt drhussien85sc@gmail.com; d Information Systems Department, College of Computer Sciences and Information Systems, Najran University Najran Saudi Arabia; e Physics Department, College of Science, Imam Mohammad Ibn Saud Islamic University (IMSIU) Riyadh 11623 Saudi Arabia

## Abstract

In this study, the biosensing capabilities of conventional and hybrid multilayer structures were theoretically examined based on surface plasmon resonance (SPR). The transfer matrix method is adopted to obtain the reflectance spectra of the hybrid multilayer structure in the visible region. In this regard, the considered SPR sensor is configured as, [prism (CaF_2_)/Al_2_O_3_/Ag/Al_2_O_3_/2D material/Al_2_O_3_/Sensing medium]. Interestingly, many optimization steps were conducted to obtain the highest sensitivity of the new SPR biosensor from the hybrid structure. Firstly, the thickness of an Al_2_O_3_ layer with a 2D material (Blue P/WS_2_) is optimized to obtain an upgraded sensitivity of 360° RIU^−1^. Secondly, the method to find the most appropriate 2D material for the proposed design is investigated to obtain an ultra-high sensitivity. Meanwhile, the inclusion of black phosphorus (BP) increases the sensor's sensitivity to 466° RIU^−1^. Thus, black phosphorus (BP) was obtained as the most suitable 2D material for the proposed design. In this regard, the proposed hybrid SPR biosensing design may pave the way for further opportunities for the development of various SPR sensors to be utilized in chemical and biomedical engineering fields.

## Abstract 2

A systematic view of the proposed hybrid SPR sensor.

## Graphical abstract

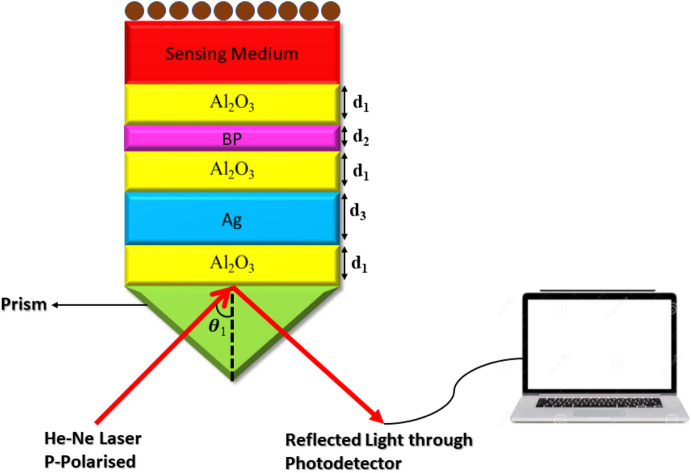

## 1. Introduction

Nowadays, surface plasmon resonance (SPR) based optical techniques are very popular. They are progressively emerging as a new research field. The SPR techniques are significant, accurate, and rapid methods due to their meaningful utilization for sensing and detecting of many chemical and biological materials. Meanwhile, SPR designs provide significant contributions through the detection and monitoring in many medical and chemical applications such as, bacteria, RNA, DNA, gases, medical diagnostics, and food technology.^1–4^ In particular, SPR-based biosensors are versatile and highly sensitive sensors due to their label-free platform, and stable and real-time detection capabilities.^4^ In most of the SPR sensors, a sample either in the solid or liquid state is examined directly without processing the sample further before examination based on the attenuated total reflection (ATR).^5^ In the ATR technique, the surface plasmon waves are stimulated along the interface between the metal and the dielectric layers of the structure due to collective oscillations of free electrons at their interfaces. Therefore, the creation of transverse magnetic (TM) radiations called surface plasmons could be introduced.^5^ Since direct exposure to a 3D beam could not excite surface waves, one can utilize techniques like grating coupling, Kretschmann configuration, and fiber coupling to excite such types of surface waves.^6^ Researchers have suggested that the Kretschmann configuration is one of the best ways to couple light from a prism to a metallic layer. Moreover, the Kretschmann configuration also provides a better signal-to-noise ratio, which allows the surface plasmons to penetrate the sensing medium for high interactions with the analyte.^7^ In this context, the phase matching between the TM polarization of the incident light and free electrons of the metal surface leads to the creation of surface plasmon waves (SPWs). These waves could act as an indicator to determine the refractive index (RI) changes of an analyte due to the interaction between biomolecules and the surface of the sensing medium.^8^ Interestingly, the matching between wave vectors of the incident light and SPWs resonantly couples the incident light with surface plasmons.^8^ This coupling results in a strong localization of the electric field called SPR.^9^ The change in the refractive index of the analyte alters the wave vector associated with the surface plasmon, which is sensed with the help of the reflectance of the TM polarized light.^8,9^ The incident angle, which corresponds to the minimum reflectance is called the resonance angle, and it is very sensitive to any change in the RI of the sensing medium.^8–10^ Therefore, the change in the RI of the sensing medium results in a corresponding change in the reflectance dip.^10^ This remarkable property of surface plasmons is used in designing various SPR sensors. The sensing performance of SPR sensors can be easily obtained with the help of the reflectance spectrum. The conventional SPR sensors have poor sensitivity and, therefore, SPR-based sensitive biosensors are in demand. Thus, many researchers introduced some novel methods and materials by which the sensitivity of the SPR biosensors can be significantly improved. For example, Almawgani *et al.* proposed a modified SPR biosensor by introducing a thin layer of black phosphorus (BP) between metal and sensing medium as an interacting medium for detecting blood concentration.^11^ Similarly, Rikta *et al.* proposed a heterostructure SPR biosensor, which is composed of an additional layer of Alfa-tin selenide and phosphorene; these two materials are placed between a metallic layer of gold and a sensing medium to obtain a maximum sensitivity of 96.43° RIU^−1^.^12^ In the same vein, Wu *et al.* and Shivangani *et al.* have explained how hybrid biosensing structures based on guided SPR waves can be used instead of conventional SPR biosensors to achieve higher sensitivity.^13,14^

In recent years, scientific developments in material science provided an opportunity of discovering engineered 2D materials with extraordinary optical and electrical characteristics.^15–17^ Today, researchers employ 2D materials like graphene, dichalcogenides, and black phosphorus (BP) in designing various SPR biosensors for the detection of biomolecules.^18^ The presence of 2D nonmaterial in the SPR biosensors improves the interactions of biomolecules of an analyte. Moreover, the 2D inorganic nonmaterial can be easily fabricated by using an electrospinning technique due to its low fabrication cost, safety, and continuous production advantages.^18–20^ Meanwhile, BP as a 2D nanomaterial has emerged as a promising candidate for SPR-based biosensing applications due to its mechanical and physical properties.^19^ Moreover, one of the remarkable prosperities of the BP is the sp^3^ hybridization due to its puckered lattice structure.^21^ The in-plane anisotropy of phosphorene makes BP the most appropriate for designing tunable biosensing devices.^22^ Additionally, the saturable absorptive property of BP makes it highly suitable to be used in biophotonics sensing applications. Furthermore, the exposure of the BP to air does not have any impact on its stability, making it highly suitable as well to be utilized in SPR biosensors.^22,23^

All the above-mentioned studies have provided useful insights into designing a hybrid SPR biosensor with enhanced sensitivity.^11–23^ In this work, conventional and hybrid SPR biosensing designs were examined. Notably, a metallic layer of silver (Ag) is considered in all the SPR designs. These designs are discussed in the subsequent sections of this paper because the dip of the SPR reflectance curve is the narrowest in SPR sensors, which are composed of Ag. As such, this Ag layer will improve sensitivity.^24^ But due to the solidity problem associated with the Ag layer, the performance of the SPR sensor is compromised, in addition to oxidation and corrosion-related issues. Therefore, a protecting layer must be used above the Ag layer to achieve a favorable performance.^25^ Therefore, a dielectric layer of aluminum oxide (Al_2_O_3_) is used as a protecting layer to overcome some issues related to Ag. Properties, such as corrosion resistance, higher ductility, and hardness make Al_2_O_3_ a promising material to be applied over mechanical areas and photonic devices due to its low refractive index and enhanced transparency. The thickness of the Al_2_O_3_ material layer is optimized by ensuring the maximum penetration of surface plasmons into the sensing medium, which is so critical for designing an SPR biosensor. In this regard, we believe that the thermal evaporation technique could be suitable for the experimental feasibility of this designed sensor. In this regard, a nanolayer of Ag metal is deposited over the Schott Lithotec-CaF_2_ prism surface. Furthermore, a thin layer of Al_2_O_3_ is grown through the chemical vapor deposition technique.^26–28^ Finally, the proposed hybrid SPR sensor can be realized in the form of a single SPR chip prepared for biosensing measurements by connecting the SPR chip through the required experimental equipment.

This paper is organized into five subsections. The first section involves an introduction to the SPR biosensor. The architecture of the proposed SPR biosensor is presented in Section 2. The mathematical framework of this work is discussed in Section 3. The results and discussion about the proposed hybrid SPR sensor are provided in Section 4. Finally, the conclusion is elucidated in Section 5.

## 2. Architecture of the proposed SPR biosensor

In this study, the biosensing capabilities of various hybrid waveguide structures, which are composed of different layers of materials, such as Al_2_O_3_, silver (Ag), and 2D materials are studied. These materials were prepared on a Schott Lithotec-CaF_2_ prism. The sensing medium (SM) is placed on the top surface through all of the suggested structures so that they can easily be loaded with the samples under investigation. Al_2_O_3_ is selected as a dielectric material for the proposed hybrid biosensing design because it is one of the well-known dielectric materials. In addition, it is widely used in the fabrication of optical and mechanical devices due to its lower refractive index, high transparency, hardness, and higher ductility, as well as its corrosion-resistant properties. Here, a wavelength of 632.8 nm is considered as the operating wavelength of the designed SPR biosensor. Meanwhile, the refractive index of Al_2_O_3_ at this wavelength is equivalent to 1.77. Furthermore, a detailed optimizing procedure is considered to choose the most suitable 2D material through our designed biosensor. Notably, 2D nanomaterials have been recently used for improving the performance of SPR-based sensors due to their unique electrical and optical properties. Also, they have high elasticity and excellent thermal conductivity, mechanical conductivity, optical transparency, and a high surface area-to-volume ratio.

The biosensing properties of all of the hybrid structures within the scope of this work are based on the SPR principle. In this work, the most common Kretschmann SPR geometry has been applied for investigating analytes of the refractive index 1.33 with a variation of 0.01.

In this regard, Table 1 presents a brief survey for all of the suggested designs regarding our SPR biosensor. Design-1 consists of a prism adjacent to the Ag layer, which belongs to the conventional SPR sensor. The waveguide (WG) structure like design-2 and design-3 is introduced by adding a dielectric layer of Al_2_O_3_ to either side of a metallic layer of design-1. The waveguide structure excites weakly the guided modes due to the coupling between the SPR modes and WG modes. The substructures design-4 and design-5 consist of dielectric–metal–dielectric (DMD) and metal–dielectric–metal (MDM) sequences, respectively. These substructures are used to produce long-range surface plasmons (LRSPs) of lower absorption losses due to the coupling of the SPR and WG modes.^11–23^ However, hybrid multilayer waveguide structures like design-6 to design-8 support the coupling of both the LRSPs and the dielectric waveguide (DWG) modes, which are responsible for creating stronger evanescent field strengths and more penetration depths. The biosensing capabilities of biosensors that are composed of hybrid multilayer structures can be further optimized by coating a dielectric thin film on the metal surface to build guided-wave surface plasmon resonance (GWSPR) based biosensors. In this study, three hybrid SPR structures with some other conventional SPR-based biosensing designs were examined. The structural details of each of the layers of design-1 to design-8 are listed in Table 2.

**Table 1:** Structural details of various conventional SPR and hybrid multilayer sensors

Structural design	Details of SPR sensor
Design 1	Prism/Ag/sensing medium
Design 2	Prism/Al_2_O_3_/Ag/sensing medium
Design 3	Prism/Ag/Al_2_O_3_/sensing medium
Design 4	Prism/Al_2_O_3_/Ag/Al_2_O_3_/sensing medium
Design 5	Prism/Ag/Al_2_O_3_/Ag/sensing medium
Design 6	Prism/Al_2_O_3_/Ag/Al_2_O_3_/2D/sensing medium
Design 7	Prism/Al_2_O_3_/2D/Al_2_O_3_/Ag/Al_2_O_3_/sensing medium
Design 8	Prism/Al_2_O_3_/Ag/Al_2_O_3_/2D/Al_2_O_3_/sensing medium

**Table 2:** Structural details of each layer of the proposed hybrid biosensing design at wavelength 632.8 nm

S. no.	Material used	Refractive index	Thickness (nm)	References
1	CaF_2_ prism	1.43289	—	29
2	Ag	0.05626 + 4.2776*i*	45	30
3	Al_2_O_3_	1.77	3	31
4	Blue P/WS_2_	2.48 + 0.17*i*	0.75	32
5	Sensing medium	1.33 to 1.34	—	32

## 3. Mathematical framework

**Fig. 1:**
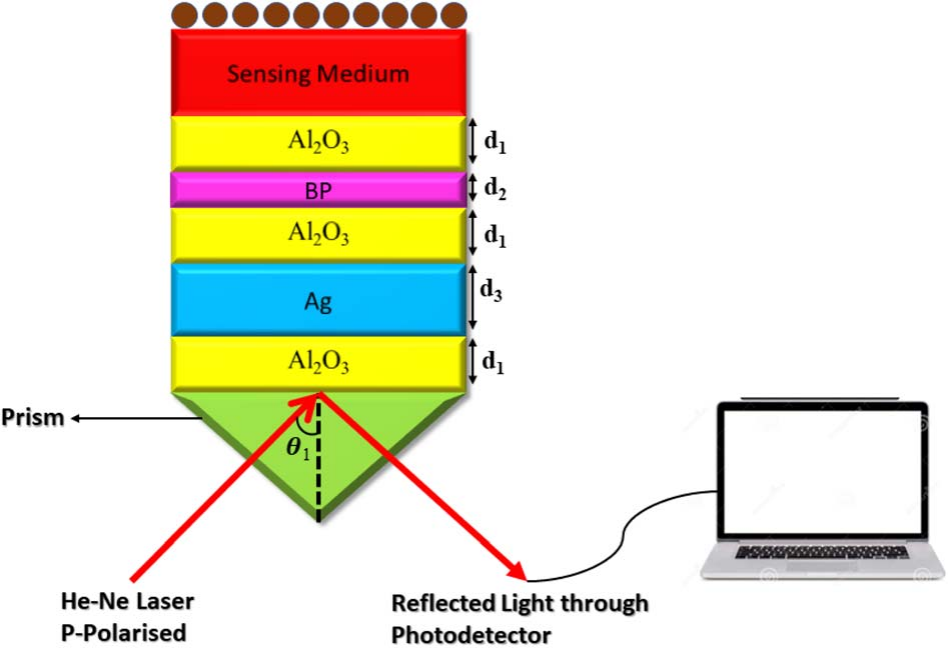
A systematic view of the proposed hybrid SPR sensor.

This section discusses the transfer matrix approach^33–36^ used in the present work to obtain the reflectivity of various conventional and hybrid multilayer designs. The considered layers are sandwiched between the Schott Lithotec-CaF_2_ prism and sensing medium to excite SPR. This method is easy, accurate and simple for calculating the reflectivity of structures composed of multiple thin film layers without using any approximation. In this context, all of the thin film layers of the structures considered in this work are arranged along the *z*-axis, as shown in Fig. 1. The thickness and refractive index of the *i*th layer of the designed structure are represented by *d*_*i*_ and *n*_*i*_, respectively. Then, a TM polarized light from a He–Ne laser is entered into the structure through the Schott Lithotec-CaF_2_ prism at an angle *θ*_1_. The tangential components of electric and magnetic fields at the interface of the *i*th layer of the structure are connected *via* a transfer matrix as follows:^6,8,33–36^1
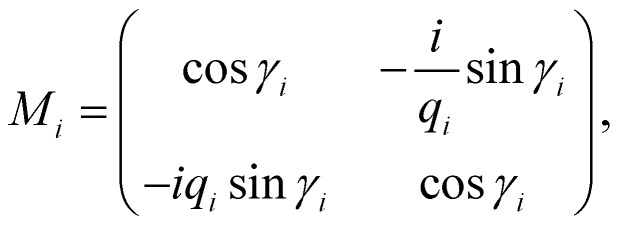
Here, 
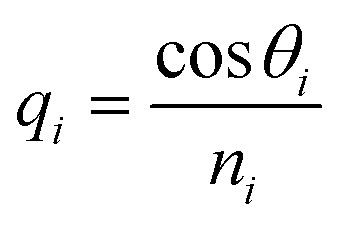
 and 
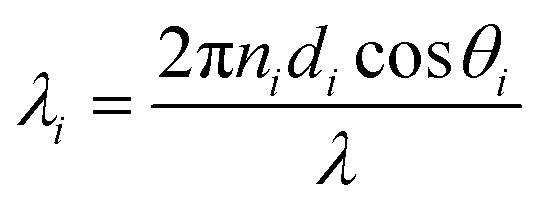
. In this regard, *n*_*i*_, *d*_*i*_, and *θ*_*i*_ describe the refractive index, thickness, and angle of incidence, respectively through a specified layer *i* of the considered structure. Then *λ* defines the wavelength of the incident electromagnetic wave. Now, one can apply Snell's law to get the value of cos *θ*_*i*_ in terms of angle of incidence as, 
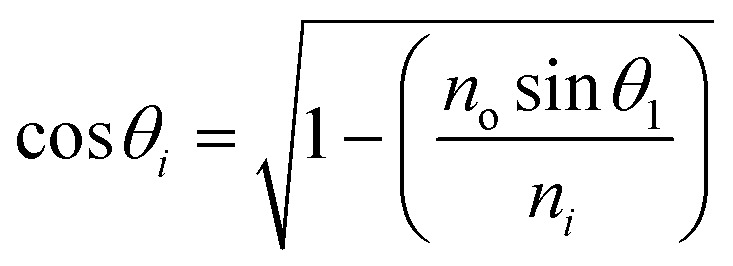
. Here, *n*_o_ is the refractive index of the ambient medium. The total transfer matrix, connecting the electric and magnetic fields of the incident and exit medium can be written as:^35^2
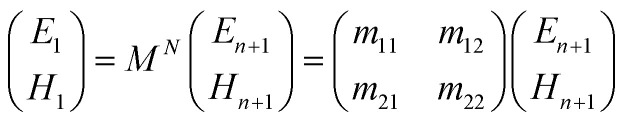
Here, the elements of the transfer matrix for the whole structure are represented as *m*_11_, *m*_12_, *m*_21,_ and *m*_22_. Thus, the reflection coefficient *r*_p_ of the whole structure is described as:^33–36^3
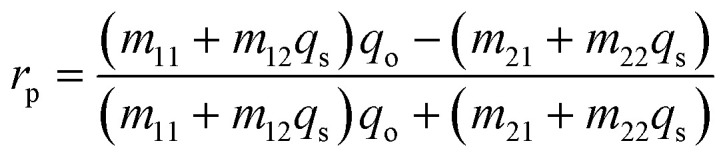


Such that, *q*_o_ and *q*_s_ are the incident (*z* < 0) and exit (*z* > *L*) media of the structure of length *L*. For TM wave 
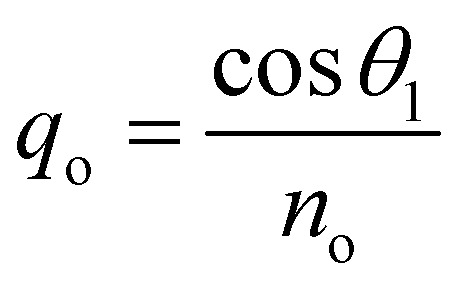
 and 
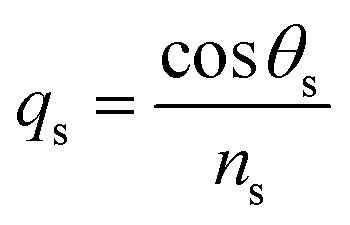
. The refractive indices of incident and exit media are represented by *n*_0_ and *n*_s_, respectively. Then, the reflectivity *R*_P_ of the SPR structure is given by:^33–36^

4 *R*_P_ = |*r*_P_|^2^.

## 4. Results and discussion

**Fig. 2:**
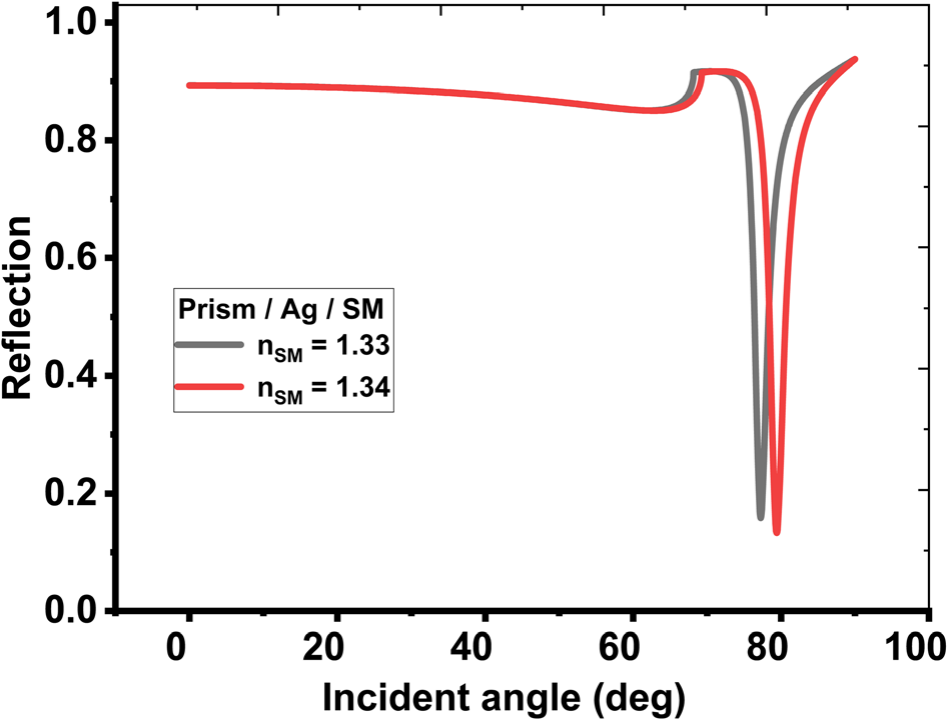
Resonance angle-dependent reflectance of conventional SPR design (prism/Ag/sensing medium) loaded with a sample of refractive index 1.33 and 1.34. Reflectance spectra show two solid line curve colors corresponding to the sensing medium of refractive index 1.33 and 1.34, respectively.

**Fig. 3:**
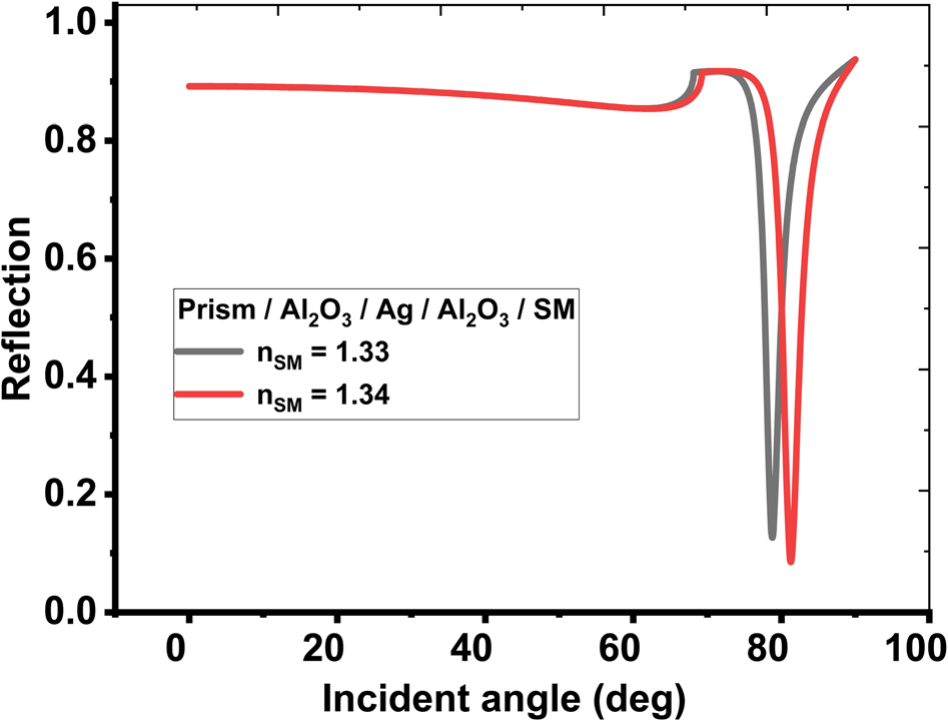
Resonance angle-dependent reflectance of modified conventional SPR design composed of prism/Al_2_O_3_/Ag/Al_2_O_3_/sensing medium. Reflectance spectra show two solid line curves corresponding to the sensing medium of refractive index 1.33 and 1.34, respectively.

**Fig. 4:**
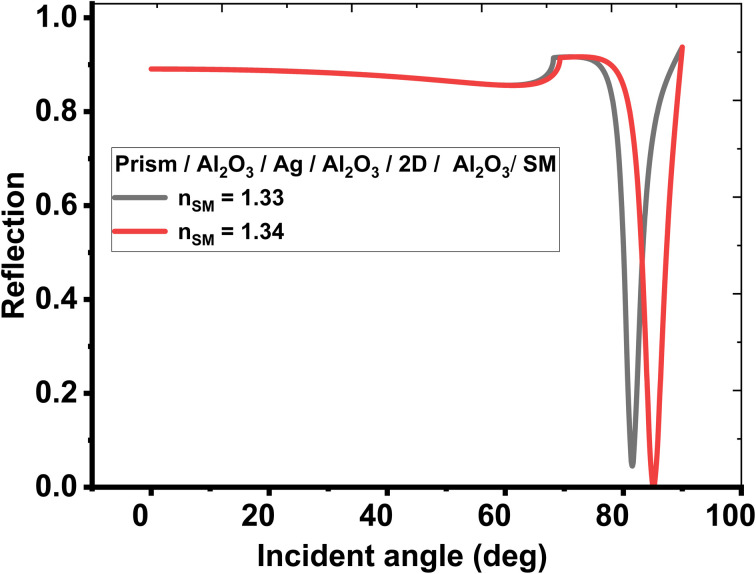
Resonance angle-dependent reflectance of hybrid multilayer design (prism/Al_2_O_3_/Ag/Al_2_O_3_/2D/Al_2_O_3_/sensing medium) loaded with a sample of refractive index 1.33 and 1.34. Reflectance spectra show two solid line curves corresponding to the sensing medium of refractive index 1.33 and 1.34, respectively.

First, the biosensing properties of various conventional and hybrid multilayer structures (see Table 1) were examined based on SPR by recording the change in the resonance angle of the reflectance dip due to the variation in the refractive index of the sensing medium from 1.33 to 1.34. The refractive indices and thicknesses of the different layers used in the conventional and hybrid multilayer designs are described in Table 2. Moreover, in all the hybrid multilayer designs, blue phosphorous-tungsten disulfide (Blue P/WS_2_) material has been randomly chosen as a 2D nanomaterial to obtain a larger variation in the resonance angle (Δ*θ*) corresponding to change in the refractive index of the sensing medium from 1.33 to 1.34. Additionally, the angle-dependent reflectivity of conventional SPR structures prism/Ag/sensing medium, prism/Al_2_O_3_/Ag/Al_2_O_3_/sensing medium, and prism/Al_2_O_3_/Ag/Al_2_O_3_/2D/Al_2_O_3_/sensing medium loaded with the analyte of refractive index variation (Δ*n*) 0.01 are plotted in Figs 2, 3, and 4, respectively.

Interestingly, Table 3 provides a brief description to the role of Al_2_O_3_ layer in improving the angular shift in the resonant angle regarding the change in the refractive index of the analyte. This enhancement can be attributed to the addition of only a dielectric layer adjacent to the metal layer to the conventional SPR sensor to enable an SPP mode.

**Table 3:** The angular shift of reflectance dips of conventional and hybrid multilayer designs corresponding to variation in the refractive index of sensing medium from 1.33 to 1.34

S. no.	Structural details	The angular shift in resonant angle (Δ*θ*°)
1	Prism/Ag/sensing medium	2.16
2	Prism/Al_2_O_3_/Ag/sensing medium	2.18
3	Prism/Ag/Al_2_O_3_/sensing medium	2.49
4	Prism/Al_2_O_3_/Ag/Al_2_O_3_/sensing medium	2.51
5	Prism/Ag/Al_2_O_3_/Ag/sensing medium	2.40 (like a capacitor)
6	Prism/Al_2_O_3_/Ag/Al_2_O_3_/2D/sensing medium	2.78
7	Prism/Al_2_O_3_/2D/Al_2_O_3_/Ag/Al_2_O_3_/sensing medium	2.52
8	Prism/Al_2_O_3_/Ag/Al_2_O_3_/2D/Al_2_O_3_/sensing medium	3.60 (best)

In fact, the metal–dielectric–metal (Ag/Al_2_O_3_/Ag) or substructures dielectric–metal–dielectric (Al_2_O_3_/Ag/Al_2_O_3_) can generate long-range surface plasmons (LRSPPs) due to the coupling of the WG and SPP modes. The LRSPP forms stronger electric field strengths accompanied by lower absorption losses. The structure Ag/Al_2_O_3_/Ag acts like a capacitor so that most of the electric field is concentrated in the dielectric layer and a small portion of this field can reach the sensing medium. Therefore, the sensitivity of the structure Al_2_O_3_/Ag/Al_2_O_3_ is higher compared to Ag/Al_2_O_3_/Ag. Accordingly, such modifications in the conventional SPR biosensing designs yielded further improvements in the angular shift in the reflectance dip corresponding to a change in the refractive index of the sensing medium.

Next, one additional layer of 2D Blue P-WS2 material has been added between the dielectric layer and the sensing medium of the structure (Al_2_O_3_/Ag/Al_2_O_3_/2D). This structure improved the angular shift in the reflectance dip to 2.78° because incorporating the 2D Blue P-WS_2_ material has enhanced the SPR and provided further resistance to corrosion.

Likewise, two further modifications were also proposed in the structure to investigate new configurations like, (Al_2_O_3_/2D/Al_2_O_3_/Ag/Al_2_O_3_) and (Al_2_O_3_/Ag/Al_2_O_3_/2D/Al_2_O_3_), respectively. Here, the second configuration, (Al_2_O_3_/Ag/Al_2_O_3_/2D/Al_2_O_3_) provides the best angular shift of 3.60° as described at sr. no. 8 of Table 3. Finally, the angle-dependent reflectance of the structures defined at sr. no. 1, 4, and 8 are plotted in Figs. 2, 3, and 4, respectively. All these figures show two resonant peaks that correspond to the change in the refractive index of sensing medium from 1.33 to 1.34, respectively. In this regard, the inclusion of three layers of Al_2_O_3_ through the proposed sensor provides the largest shift in the angle of incidence with the change in the refractive index of the analyte (as investigated in table (3)). Therefore, the sensitivity of the designed sensor could be significantly increased to reach 360° RIU^−1^.

### 4.1 Optimization of the dielectric layer's thickness used in the hybrid multilayer design (prism/Al_2_O_3_/Ag/Al_2_O_3_/2D/Al_2_O_3_/sensing medium)

In this section, an improvement has been carried out to the shift in the resonant angle of the hybrid design, which is composed of (prism/Al_2_O_3_/Ag/Al_2_O_3_/2D/Al_2_O_3_/sensing medium). The optimization of the Al_2_O_3_ material's thickness is one of the requirements in the development of biosensing devices constructed of a hybrid multilayer structure. Table 4 illustrates the shift in the resonant angle of the hybrid design composed of (prism/*n*/Ag/Al_2_O_3_/2D/Al_2_O_3_/sensing medium) corresponding to the dielectric material of different thicknesses. Other structural parameters of this design are similar, as provided in Table 2.

**Table 4:** Shift in resonance angle (Δ*θ*°) corresponding to Δ*n* = 0.01 with dielectrics of different thicknesses and refractive indices

The thickness of the dielectric layer in nm	Shift in resonance angle (Δ*θ*°) corresponding to Δ*n* = 0.01 with dielectrics of refractive indices
0	2.32
1	2.59
2	3.02
3	3.60 (best)
4	2.21

Now, the shift in Δ*θ*° is recorded, which corresponds to a change in the refractive index of sensing medium Δ*n* = 0.01 by randomly varying the thickness of the dielectric layer of the suggested configuration, as illustrated in Table 4. This process will help in optimizing the thickness of the dielectric layer used in the design to obtain further improvements in Δ*θ*°, which can, in turn, improve the sensing capabilities of the hybrid design. Based on Table 4, it is observed that the Δ*θ*° value increased up to a maximum value, then it decreased with increasing the thickness of the dielectric layer and light underwent multiple reflections inside the dielectric layer. At the optimum thickness of the dielectric layer, the constructive interferences of light can maximize the transfer of energy, resulting in a strong SPR.^11^ Therefore, the thickness of Al_2_O_3_ layers utilized in the hybrid design should be 3 nm. Notably, the maximum shift in the resonant angle was investigated at this value as clarified in Table 4.

### 4.2 Optimization of refractive index and thickness of 2D material used in the hybrid multilayer design prism/Al_2_O_3_/Ag/Al_2_O_3_/2D/Al_2_O_3_/sensing medium

In this subsection, we have discussed the optimum 2D material that could be included through our designed SPR biosensor. In fact, a maximum shift of 3.60° in the resonant angle was achieved by considered a 2D material of Blue P/WS_2_. Now, more 2D materials of different thicknesses were used instead of the Blue P/WS_2_ 2D material layer in the proposed hybrid multilayer design to obtain further improvements in an angular shift of reflectance dip. The details of all of the suggested 2D materials with their refractive indices and thicknesses at wavelength 632.8 nm are listed in Table 5.

**Table 5:** Shift in resonance angle (Δ*θ*°) corresponding to Δ*n* = 0.01 with different 2D materials used in the hybrid design composed of prism/Al_2_O_3_/Ag/Al_2_O_3_/2D/Al_2_O_3_/sensing medium. The refractive indices of all 2D materials are calculated at wavelength 632.8 nm

Details of 2D materials	Refractive index at wavelength 632.8 nm	The thickness of 2D layers (nm)	Δ*θ*
BlueP/WS2	2.48 + 0.17*i*	0.75	3.60
Graphene oxide (GO)	1.2728 + 0.0039*i*	2.55	3.2
MXene (Ti_3_C_2_T_*x*_)	2.38 + 1.33*i*	0.993	3.26
Antimonene (Sb)	1.4 + 1.3*i*	0.5	3.7
Graphene (G)	3.0 + 1.149*i*	0.35	4.05
Black phosphorus (BP)	3.5 + 0.01*i*	0.53	4.75 (best)

The data analysis, as presented in Table 5, showed the angular shift in the reflectance dip corresponding to Δ*n* = 0.01 of the proposed hybrid multilayer design. In this regard, the dependence on other types of 2D materials like antimonene (Sb) and black phosphorus (BP) could provide a shift of 4.05° and 4.75° in the resonant angle, respectively. Therefore, the performance of the considered SPR biosensor can be significantly improved. Meanwhile, the choice of BP represents the optimum one towards a higher shift in the resonant angle and the sensor performance as well. Thus, the optimum design of our suggested SPR biosensor will be configured as, prism/Al_2_O_3_/Ag/Al_2_O_3_/BP/Al_2_O_3_/sensing medium. Then, the layers' thicknesses of the optimized hybrid multilayer design are listed in Table 6.

**Table 6:** Thicknesses of layers used in the optimized hybrid multilayer design prism/Al_2_O_3_/Ag/Al_2_O_3_/BP/Al_2_O_3_/sensing medium

Position of layers from top	Material used	Thickness (nm)
Layer # 1	Prism	Semi-infinite
Layer # 2	Al_2_O_3_	3 nm
Layer # 3	Ag	45 nm
Layer # 4	Al_2_O_3_	3 nm
Layer # 5	BP	0.53 nm
Layer # 6	Al_2_O_3_	3 nm
Layer # 7	Sensing medium	Semi-infinite

Actually, BP is a direct bandgap semiconductor material that consists of a single layer with a honeycomb lattice.^6^ Moreover, BP has a high real dielectric constant and a very low extinction coefficient, which can be used to minimize the losses.^3,6^ Additionally, BP exhibits a high molecular adsorption energy, which makes it ideal for trapping biomolecules.^11^ This feature makes the BP suitable for strong SPR coupling. Therefore, introducing the BP can achieve high sensor performance of the proposed sensor compared to other 2D materials.

### 4.3 Evaluation of the performance of optimized hybrid multilayer structure

**Fig. 5:**
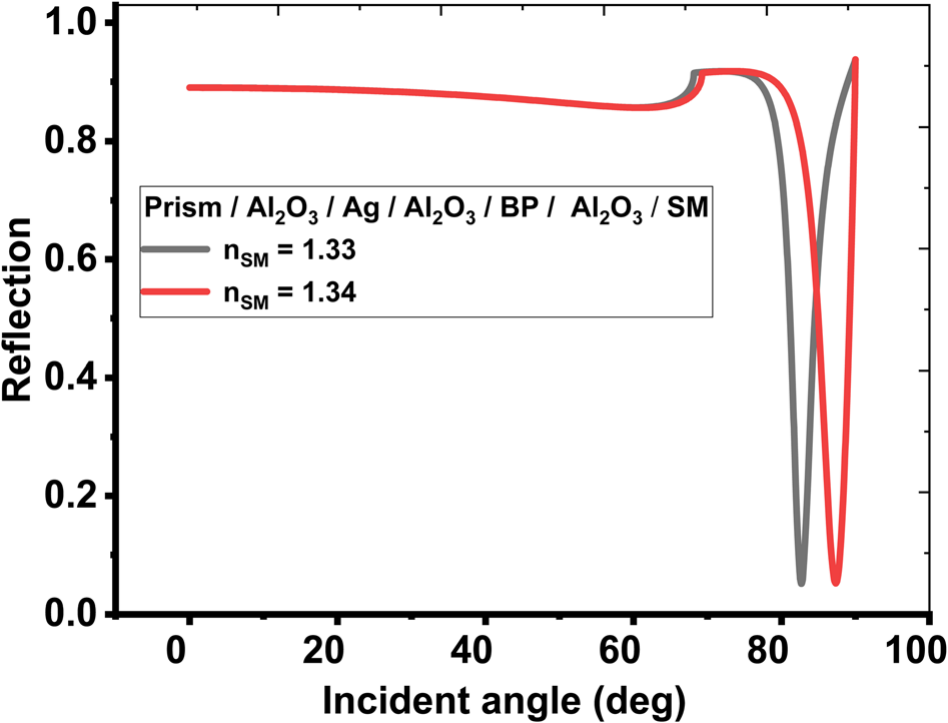
Resonance angle-dependent reflectance of optimized hybrid multilayer design composed of prism/Al_2_O_3_/Ag/Al_2_O_3_/BP/Al_2_O_3_/sensing medium loaded with a sample of refractive index 1.33 and 1.34.

This section discusses the performance of the optimized hybrid multilayer structure, which is composed of prism/Al_2_O_3_/Ag/Al_2_O_3_/BP/Al_2_O_3_/sensing medium, as shown in Fig. 1. The performance of any SPR-based biosensing design is dependent on some variables, which are interlinked with one another. These interlinked variables are used for the performance evaluation of SPR-based sensors in terms of sensitivity, the figure of merit, quality factor, limit of detection, and limit of qualification. Moreover, the incident angle-dependent reflectivity plot of the optimized hybrid multilayer structure composed of prism/Al_2_O_3_/Ag/Al_2_O_3_/BP/Al_2_O_3_/sensing medium, as shown in Fig. 5, plays a key role in determining the performance of the proposed SPR sensor. It shows a maximum angular shift in reflectance dip of 4.75° corresponding to a change in the refractive index of the sensing medium from 1.33 to 1.34. This maximum change in the angular position of the reflectance dip can be attributed to the superposition of the surface plasmon waves (SPWs). Under these circumstances, the proposed optimized hybrid multilayer structure could detect the minute change in the refractive index of the sensing medium and, thus, becomes highly sensitive.

**Fig. 6:**
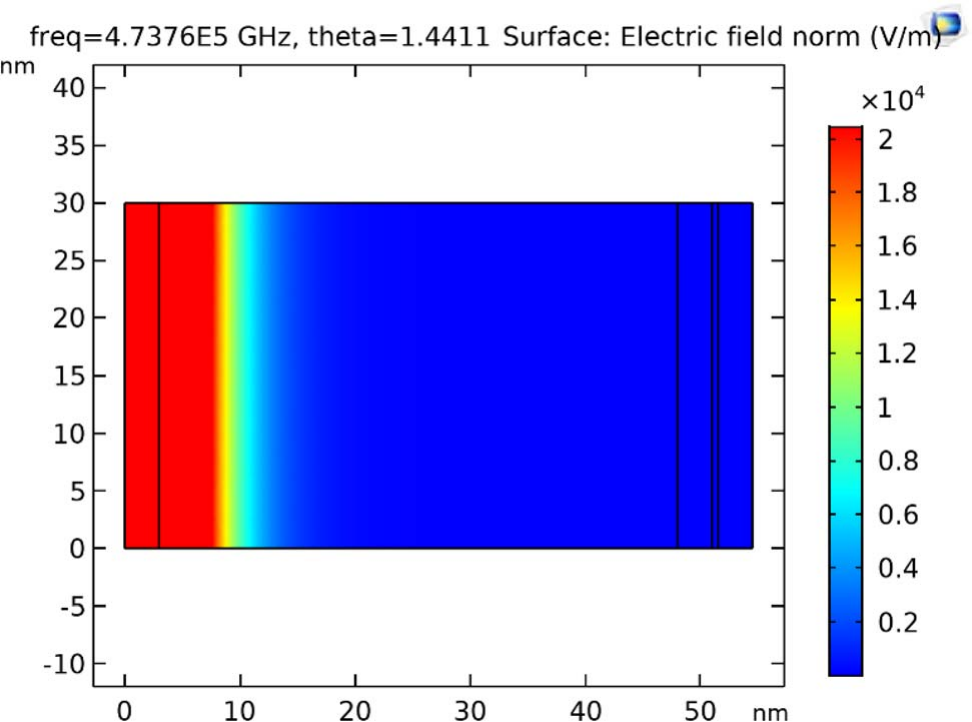
The electric field distribution through the designed SPR biosensor.

Then, Fig. 6 show the electric field distribution through the designed SPR biosensor at the optimum condition in the case of the resonance angle (82.68°). Here, the field localization varies along the different layers of the considered structure.

### 4.4 Sensitivity (*S*)

The change in the angular position of the reflectance dip (Δ*θ*) for the change in the refractive index of the sensing medium (Δ*n*) is defined as a sensitivity of the proposed design. Mathematically, it is defined as:^24^5
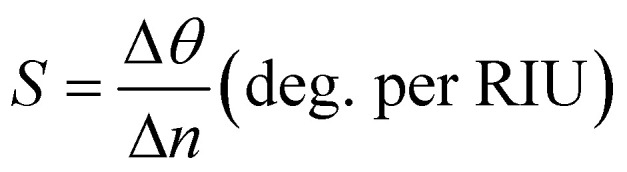


### 4.5 Figure of merit (FoM)

The performance of the SPR sensor is characterized by a quantity called the figure of merit. Mathematically, it is defined as:^14^6
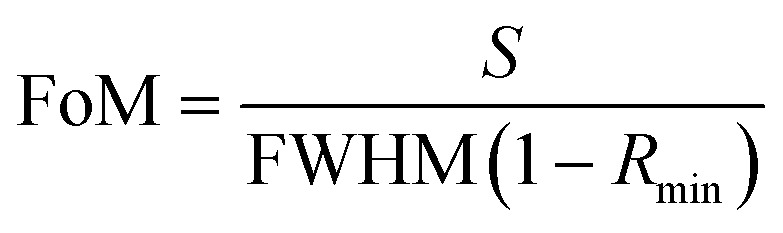
Here, *R*_min_ is the reflectivity of the reflectance dip corresponding to the resonance angle. To calculate the full-width half maximum (FWHM) of the reflectance dip, the change in the incident angle must be calculated at the midway point of the reflectance dip.

### 4.6 Quality factor (QF)

The quality factor of the SPR sensor depends on the incident angle at which reflectance becomes minimum (*R*_min_), called the resonance angle or the SPR angle (*θ*_SPR_) and FWHM. It is defined as:^24^7
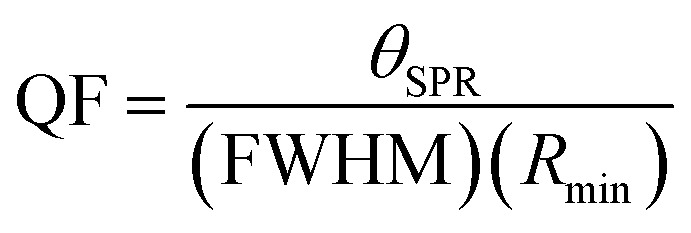


After optimizing the proposed hybrid multilayer biosensing design composed of prism/Al_2_O_3_/Ag/Al_2_O_3_/BP/Al_2_O_3_/sensing medium loaded with sensing medium of refractive indices 1.330, 1.332, 1.334, 1.336, 1.338, and 1.340 one at a time, the performance of the optimized structure loaded with different samples has been evaluated by calculating the parameters S, FoM, and QF as listed in Table 7.

**Table 7:** Performance evaluation of the optimized hybrid multilayer biosensing structure prism/Al_2_O_3_/Ag/Al_2_O_3_/BP/Al_2_O_3_/sensing medium loaded with different samples

RI of sensing medium (*n*_SM_)	Resonance angle (deg.)	*R*_min_ at *θ*_SPR_	FWHM (deg.)	*S* (deg. per RIU)	FoM (RIU^−1^)	QF (RIU^−1^)
1.330	82.68	0.059	3.70	–	–	378.74
1.332	83.38	0.045	3.81	350	96.19	486.32
1.334	84.16	0.029	3.94	370	96.71	736.56
1.336	85.05	0.014	4.05	395	98.95	1458.33
1.338	86.10	0.009	4.11	427	104.91	2252.57
1.340	87.34	0.055	4.18	466	118.04	375.80

Table 7 demonstrates that the proposed hybrid multilayer design can be used for detecting the sensing medium whose RI varies between 1.330 and 1.340. The response of the SPR reflectivity curve, as plotted in Fig. 5, is recorded in Table 7. It indicates that the change in the RI of the sensing medium from 1.330 to 1.340 caused the SPR reflectance dip to shift from 82.68° to 87.34°, resulting in a maximum sensitivity of 466° RIU^−1^, FoM of 118.04 RIU^−1^, and QF of 375.80 RIU^−1^. Thus, the performance of the proposed hybrid SPR design is more efficient compared with the design suggested by Shivangani *et al.*^14^ Next, the dependence of the SPR angle *θ*_SPR_ on the RI of the sensing medium of the proposed hybrid multilayer design has been analyzed. For this purpose, Fig. 6 is plotted, which shows how the *θ*_SPR_ increases as the RI of the medium increases to be sensed by the proposed optimized design. Fig. 6 shows that *θ*_SPR_ increased from 82.68° to 87.34° as the RI of the sensing medium changed from 1.330 to 1.340. The linear curve fitting has also been applied to the simulated data, as plotted in Fig. 6, yielding a curve fitting equation *θ*_SPR_ = 462.14*n*_SM_ − 532.1. The dashed line curve in Fig. 6 represents a linear curve fitting between *θ*_SPR_ and *n*_SM_. Here, *R*^2^ = 0.988 is the root mean square value between the linear fitting and simulated data. It indicates that *θ*_SPR_ increases linearly with an increase in n_SM_.

**Fig. 7:**
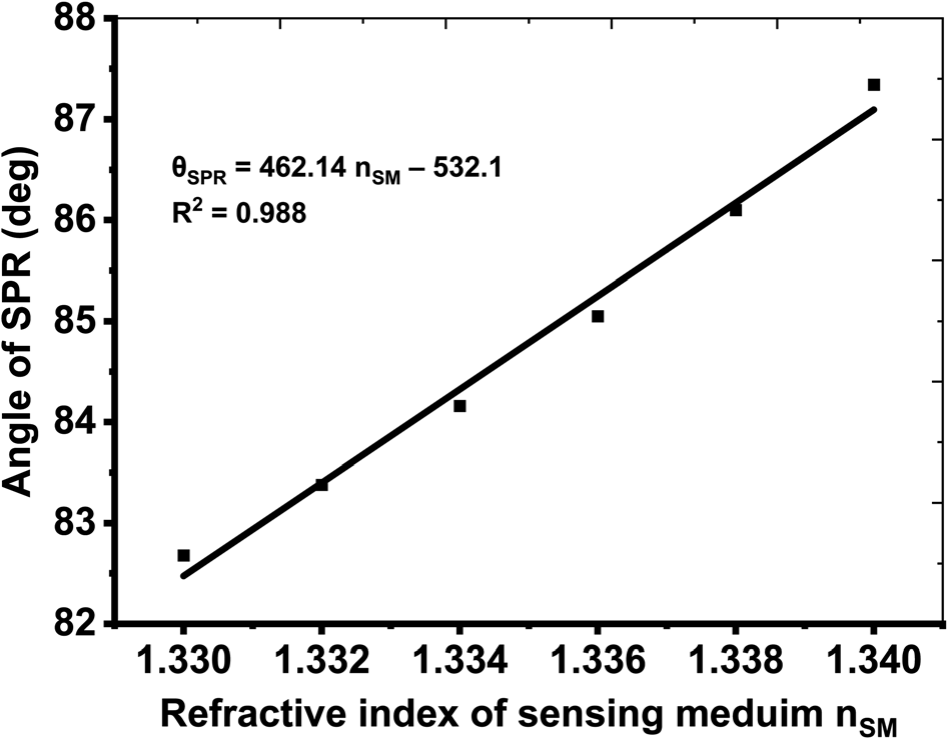
Relationship between *θ*_SPR_ and *n*_SM_. Solid dots represent the simulated data, and the dashed line represents the linear curve fitting applied to the simulated data.

**Fig. 8:**
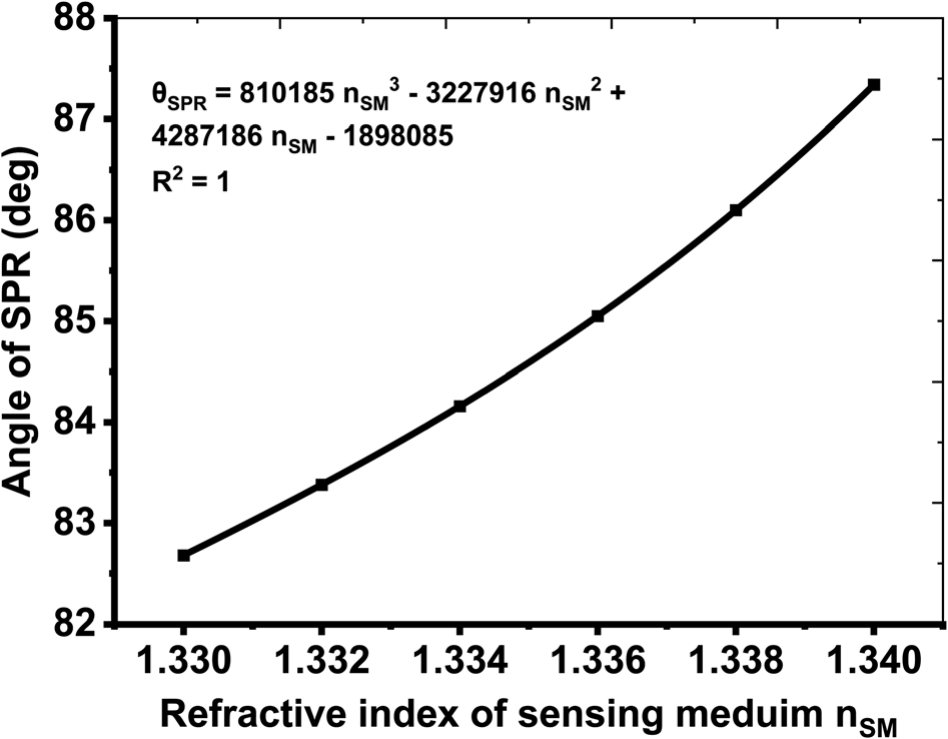
Relationship between *θ*_SPR_ and *n*_SM_. Solid dots represent simulated data, and the dashed line represents the polynomial curve fitting applied to the simulated data.

Fig. 7 can be used to find the detection limit and limit of qualification of the optimized hybrid biosensing design, in addition to Table 7. The parameters detection limit (DL) and limit of qualification (LoQ) of any SPR-based biosensors are defined as:^14,24^8
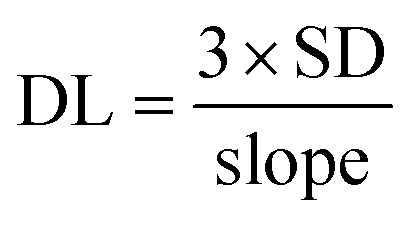
9
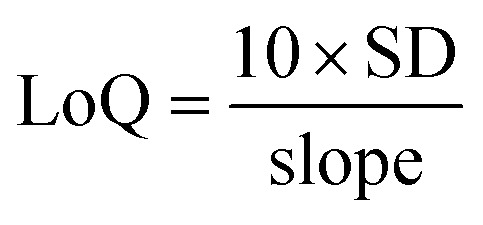
Here, the slope of the line, as plotted in Fig. 6, is 462.14 and the standard deviation (SD) of the refractive indices of the samples under consideration (see Table 7) is 0.023886654. The numeric values of slope and SD are used to obtain DL and LoQ as 0.000170567 and 0.023886654 with the help of eqn (8) and (9), respectively. Moreover, the polynomial curve fitting of the 2nd order has also been applied to *θ*_SPR_ and *n*_SM,_ yielding Fig. 8. It gives the polynomial curve fitting eqution *θ*_SPR_ = 810185*n*_SM_^3^ − 3227916*n*_SM_^2^ + 4287186*n*_SM_ − 1898085 with *R*^2^ = 1. Here, *R*^2^ represents the root mean square value (*R*^2^) between the polynomial curve fitting and simulated data.

Then, in Table 8, we compare our results with some of related works in this field to show the magnificent performance and sensitivity of our sensor over the previous published sensors. In this table, it was shown that the proposed sensor produced an enhanced sensitivity of 466° RIU^−1^, which is remarkably higher than most of the cited works in Table 8. Since, the highest one just get sensitivity of 350° RIU^−1^, which is less than of the obtained sensitivity by the amount of 166° RIU^−1^.

**Table 8:** Comparison of the obtained sensitivity of this work with the most recent surface plasmon resonance (SPR) biosensors

Designed structure	Sensitivity (° RIU^−1^)	Year	References
Fat concentration detection in milk employing SPR biosensor using Si and Ti_3_C_2_T_*x*_	350	2022	9
Black phosphor sheets as a SPR sensor	124	2022	11
2D-nanomaterial of graphene as SPR biosensor	161	2022	37
Titanium disilicide and black phosphorus as a SPR biosensor	195.4	2022	38
Graphene sheets as a SPR biosensor for sensing of bacteria	199.87	2022	39
SPR photodetector based on plasma layer	102.9	2021	40
SPR photodetector based on ZnO for hybridization of DNA	157	2020	41
MXene and black phosphorus SPR biosensor	190.22	2020	42
SPR biosensor using a thin layer of graphene	121.7	2019	43

## 5. Fabrication feasibility and experimental tolerance

Finally, we present in this section the fabrication feasibility regarding the our designed SPR biosensor besides the experimental tolerance. In fact, the manufacturing of any designed biosensor is crucial towards to the rising of this design through the real environment. In this regard, the optimized proposed sensor, which consists of a prism/Al_2_O_3_/Ag/Al_2_O_3_/BP/Al_2_O_3_/sensing medium structure, can be fabricated through several steps. The substrate for the sensor is the Schott Lithotec-CaF_2_ prism. Initially, an ultra-thin Al_2_O_3_ film is deposited onto the prism using the atomic layer deposition (ALD) technique at a low temperature with high-quality.^44,45^ Then, the Ag film is grown on the top of the prism/Al_2_O_3_ by utilizing the ALD method.^46,47^ Then, a second Al_2_O_3_ layer is prepared through the ALD process. Interestingly, ALD is a highly promising technique for depositing thin films of an organic–inorganic hybrid type of materials on different substrates. It offers numerous advantages and benefits, such as high-quality, good composition control, and contamination-free conditions, with excellent adhesion.^46^ The ALD process results in a uniform, smooth, and defect-free surface, without any cracks or voids, and without the development of columnar growth.^45^ The ability of ALD to deposit films with precise control at the atomic scale provides additional advantages, particularly for tailoring interfaces with great precision.^48,49^ For the 2D black phosphorus (BP) film, it can be prepared directly using chemical vapor deposition (CVD) from red phosphorus in a vacuum through the substrate.^50^ Finally, the Al_2_O_3_ can be fabricated on the prism/Al_2_O_3_/Ag/Al_2_O_3_/BP by using the ALD. For analyte detection, the target solution is injected in contact with the top surface of the proposed sensor. Meanwhile, a system of a lens, polarizer, and collimator can be utilized to direct the monochromatic light from the He–Ne laser towards the prism.^10^ Then, a photodetector is used to detect the intensity of the reflected light.

On the other hand, the fabrication tolerance is a crucial parameter that can provide a significant effect on the performance of thin films in some diverse applications. The thickness tolerance for a thin film is typically defined as a range of acceptable variations in the film's thickness. In nanophotonics, for instance, the thickness tolerance of a thin film can have a significant impact on the performance of optical devices, necessitating tight thickness tolerances. The acceptable tolerances depend on the specific application and requirements of the film. Generally, film thickness tolerances are expressed as a range, for example, ±5% of the nominal thickness. If the film thickness falls outside this range, it may not be suitable for the intended use. ALD's sub-nanometer thickness control is highly beneficial for achieving precise tailoring of interfaces with great accuracy, making it an ideal technique for fabricating thin films with tight tolerances.^51–53^ In this regard, Table 9 clarifies the role of tolerance on the shift in the resonant angle and the sensitivity of the SPR sensor as well. Here, two different values of fabrication tolerance are considered *i.e.*, ±5% and ±10%. The investigated numerical findings show that the sensor sensitivity could provide a little increase as the fabrication error receives +5%. On the other hand, the sensitivity could be decreased by 60 (° RIU^−1^) due to a fabrication tolerance of −5%. For further increase in the fabrication tolerance to +10% or −10%, the sensor's sensitivity could be significantly decreased.^53^ Therefore, a tolerance value of ±5% is accepted towards a relatively high performance of the sensing tool.

**Table 9:** The impact of fabrication tolerance on the shift in the resonant angle and the sensitivity of the SPR biosensor as well

Fabrication tolerance	Δ*θ* (°)	Sensitivity (° RIU^−1^)
Optimized	4.66	466
+5%	4.74	474
−5%	4.06	406
+10%	3.19	319
−10%	3.56	356

## 6. Conclusion

This study investigated a novel method to enhance the sensitivity of conventional SPR sensors by using a hybrid multilayer SPR sensor, which is composed of a 2D material BP. The angular sensitivity of the proposed hybrid SPR structure consisting of a 2D martial BP reached 466° RIU^−1^ when the refractive index of the sensing medium changed from 1.330 to 1.340. The FoM and QF values of the proposed hybrid SPR sensor varied from 9.19 to 118.04, and 378.74 to 2252.57/RIU, respectively. The DL and LoQ values of the design were determined with the help of the standard deviation of sensitivity of the SPR sensor (0.023886654) and the slope of the straight line (462.14), which were 0.000170567 and 0.000516871, respectively. Moreover, the fabrication tolerance could provide a significant effect on the sensitivity of the designed sensor. However, a tolerance value of ± 5% is accepted towards a relatively high performance of the sensing tool. Finally, the proposed design in this work could be promising in many areas of chemical and biomedical liquid sensing due to the simplicity of the design. It is also easy to fabricate and is characterized by high performance.

## Ethics approval

I, hereby, the corresponding author declare that the authors have thoroughly read the Journal Policy and admitted all its requirements. Specially, I declare here that this contribution is original and has not been published anywhere. I also declare that this article doesn't contain any plagiarized materials. No part of this manuscript has been introduced in any conference or published in any journal.

## Data availability

The data that support the findings of this study will be made available from the corresponding author upon reasonable request.

## Author contributions

A. M. Ahmed and H. A. Elsayed conceived of the presented idea and developed the theory. A. M. Ahmed, A. H. M. Almawgani, G. A. Ali and H. A. Elsayed. A. Mehaney performed the computations. S. Kumar Awasthi wrote the manuscript with support from H. A. Elsayed, H. Sayed, A. Mehaney and A. H. M. Almawgani. S. Kumar Awasthi, G. A. Ali and A. H. M. Almawgani visualization, administration, and funding. All authors discussed the results and contributed to the final manuscript.

## Conflicts of interest

The authors declare they have no conflicts of interests.

## Supplementary Material

## References

[cit1] Almawgani A. H., Taya S. A., Daher M. G., Colak I., Wu F., Patel S. K. (2022). Detection of glucose concentration using a surface plasmon resonance biosensor based on barium titanate layers and molybdenum disulphide sheets. Phys. Scr..

[cit2] Pal A., Jha A. (2021). A theoretical analysis on sensitivity improvement of an SPR refractive index sensor with graphene and barium titanate nanosheets. Optik.

[cit3] Singh Y., Raghuwanshi S. K. (2019). Sensitivity Enhancement of the Surface Plasmon Resonance gas sensor with Black Phosphorus. IEEE Sens. Lett..

[cit4] Uniyal A., Srivastava G., Pal A. (2023). *et al.*, Recent Advances in Optical Biosensors for Sensing Applications: a Review. Plasmonics.

[cit5] Kretschmann E., &Raether H. (1968). Radiative decay of non radiative surface plasmons excited by light. Z. Naturforsch. A.

[cit6] Raghuwanshi S. K. , Santosh Kumar S., and Singh Y., 2D Materials for Surface Plasmon Resonance-Based Sensors, CRC Press, Boca Raton, 1st edn, 2021

[cit7] Chen S., Hu S., Wu Y., Deng D., Luo Y., Chen Z. (2021). Ultrasensitive biosensor with hyperbolic metamaterials composed of silver and zinc oxide. Nanomaterials.

[cit8] Pandey P. S., Raghuwanshi S. K., Singh Y. (2022). Enhancement of the sensitivity of a surface plasmon resonance sensor using a nobel structure based on barium titanate–graphene–silver. Opt. Quantum Electron..

[cit9] Almawgani A. H., Daher M. G., Taya S. A., Mashagbeh M., &Colak I. (2022). Optical detection of fat concentration in milk using MXene-based surface plasmon resonance structure. Biosensors.

[cit10] Uniyal A., Pal A., Srivastava G. (2023). *et al.*, Surface plasmon resonance biosensor sensitivity improvement employing of 2D materials and BaTiO_3_ with bimetallic layers of silver. J. Mater. Sci.: Mater. Electron..

[cit11] Almawgani A. H., Daher M. G., Taya S. A., Olaimat M. M., Alhawari A. R., &Colak I. (2022). Detection of blood plasma concentration theoretically using SPR-based biosensor employing black phosphor layers and different metals. Plasmonics.

[cit12] Rikta K. A., Anower M. S., Rahman M. S., Rahman M. M. (2021). SPR biosensor using SnSe-phosphorene heterostructure. Sensing and Bio-Sensing Research.

[cit13] Wu L., Guo J., Dai X., Xiang Y., Fan D. (2018). Sensitivity enhanced by MoS_2_–graphene hybrid structure in guided-wave surface plasmon resonance biosensor. Plasmonics.

[cit14] Karki B., Uniyal A., Srivastava G., Pal A. (2023). Black Phosphorous and Cytop Nanofilm-Based Long-Range SPR Sensor with Enhanced Quality Factor. J. Sens..

[cit15] Pandey P. S., Singh Y., Raghuwanshi S. K. (2021). Theoretical Analysis of the LRSPR Sensor With Enhance FOM for Low Refractive Index Detection Using MXene and Fluorinated Graphene. IEEE Sensor. J..

[cit16] Mudgal N., Yupapin P., Ali J., Singh G. (2020). BaTiO_3_-graphene-affinity layer-based surface plasmon resonance (SPR) biosensor for pseudomonas bacterial detection. Plasmonics.

[cit17] Verma R., Gupta B. D., Jha R. (2011). Sensitivity enhancement of a surface plasmon resonance-based biomolecules sensor using graphene and silicon layers. Sens. Actuators, B.

[cit18] El-Amassi D. M., Taya S. A. (2017). Reflection through a parallel-plate waveguide formed by two graphene sheets. Photon. Nanostruct: Fundam. Appl..

[cit19] Duan X., Wang C., Shaw J. C., Cheng R., Chen Y., Li H. (2014). *et al.*, Lateral epitaxial growth of two-dimensional layered semiconductor
heterojunctions. Nat. Nanotechnol..

[cit20] Ji W., Zhao G., Guo C., Fan L., Deng H., Du R., Fu Q. (2021). A novel method to fabricate two-dimensional nanomaterial based on electrospinning. Compos. Appl. Sci. Manuf..

[cit21] Verma V. K., Pal S., Rizal C., Prajapati Y. K. (2022). Tunable and sensitive detection of cortisol using anisotropic phosphorene with a surface plasmon resonance technique: numerical investigation. Magnetochemistry.

[cit22] Verma A., Sharma A. K., Prajapati Y. K. (2021). Simulation and analysis of SPR-based biosensor with borophene and antimonene layers. Opt. Mater..

[cit23] Wood J. D., Wells S. A., Jariwala D., Chen K. S., Cho E., Sangwan V. K. (2014). *et al.*, Effective passivation of exfoliated black phosphorus transistors against ambient degradation. Nano Lett..

[cit24] Alotaibi M. F., Al-Hadeethi Y., Lohia P., Singh S., Dwivedi D. K., Umar A., Baskoutas S. (2022). Numerical study to enhance the sensitivity of a surface plasmon resonance sensor with BlueP/WS2-covered Al_2_O_3_-nickel nanofilms. Nanomaterials.

[cit25] Tang N., Li Y., Chen F., Han Z. (2018). In situ fabrication of a direct Z-scheme photocatalyst by immobilizing CdS quantum dots in the channels of graphene-hybridized and supported mesoporous titanium nanocrystals for high photocatalytic performance under visible light. RSC Adv..

[cit26] Borges J., Vaz F., Marques L. (2010). AlNxOy thin films deposited by DC reactive magnetron sputtering. Appl. Surf. Sci..

[cit27] Borges J., Barradas N. P., Alves E., Beaufort M. F., Eyidi D., Vaz F., Marques L. (2013). Influence of stoichiometry and structure on the optical properties of AlN_*x*_O_*y*_ films. J. Phys. D: Appl. Phys..

[cit28] Sharma A. K. (2018). Analyzing the application of silicon–silver–2D nanomaterial–Al_2_O_3_ heterojunction in plasmonic sensor and its performance evaluation. Opt. Commun..

[cit29] Mudgal N., Yupapin P., Ali J., Singh G. (2020). BaTiO_3_-graphene-affinity layer–based surface plasmon resonance (SPR) biosensor for pseudomonas bacterial detection. Plasmonics.

[cit30] Alotaibi M. F., Al-Hadeethi Y., Lohia P., Singh S., Dwivedi D. K., Umar A., Alzayed H. M., Algadi H., Baskoutas S. (2022). Numerical study to enhance the sensitivity of a surface plasmon resonance sensor with BlueP/WS2-covered Al_2_O_3_-nickel nanofilms. Nano.

[cit31] Liu N., Wang S., Cheng Q., Pang B., Lv J. (2021). High sensitivity in Ni-based SPR sensor of blue phosphorene/transition metal dichalcogenides hybrid nanostructure. Plasmonics.

[cit32] Kushwaha A. S., Kumar A., Kumar R., Srivastava S. K. (2018). A study of surface plasmon resonance (SPR) based biosensor with improved sensitivity. Phot. Nano. Fund. Appl..

[cit33] Aly A. H., Awasthi S. K., Mohamed A. M., Al-Dossari M., Matar Z. S., Mohaseb M. A., Amin A. F. (2021). 1D reconfigurable bistable photonic device composed of phase change material for detection of reproductive female hormones. Phys. Scr..

[cit34] Aly A. H., Awasthi S. K., Mohamed A. M., Matar Z. S., Mohaseb M. A., Al-Dossari M., Sabra W. (2021). Detection of reproductive hormones in females by using 1D photonic crystal-based simple reconfigurable biosensing design. Crystals.

[cit35] Aly A. H., Awasthi S. K., Mohamed D., Matar Z. S., Al-Dossari M., Amin A. F. (2021). Study on a one-dimensional defective photonic crystal suitable for organic compound sensing applications. RSC Adv..

[cit36] Awasthi S. K., Malaviya U., Ojha S. P. (2006). Enhancement of omnidirectional total-reflection wavelength range by using one-dimensional ternary photonic bandgap material. J. Opt. Soc. Am. B.

[cit37] Karki B., Uniyal A., Pal A., Srivastava V. (2022). Hemoglobin detection in blood samples using a graphene-based surface plasmon resonance biosensor. Optik.

[cit38] Karki B., Uniyal A., Pal A., Srivastava V. (2022). Advances in surface plasmon resonance-based biosensor technologies for cancer cell detection. Int. J. Opt..

[cit39] Daher M. G., Taya S. A., Colak I., Patel S. K., Olaimat M. M., Ramahi O. (2022). Surface plasmon resonance biosensor based on graphene layer for the detection of waterborne bacteria. J. Biophot..

[cit40] Taya S. A., Al-Ashi N. E., Ramahi O. M., Colak I., Amiri I. S. (2021). Surface plasmon resonance-based optical sensor using a thin layer of plasma. J. Opt. Soc. Am. B.

[cit41] Pal S., Prajapati Y. K., Saini J. P. (2020). Influence of graphene's chemical potential on SPR biosensor using ZnO for DNA hybridization. Opt. Rev..

[cit42] Srivastava A., Verma A., Das R., Prajapati Y. K. (2020). A theoretical approach to improve the performance of SPR biosensor using MXene and black phosphorus. Optik.

[cit43] Hossain M. B., Mehedi I. M., Moznuzzaman M., Abdulrazak L. F., Hossain M. A. (2019). High performance refractive index SPR sensor modelling employing graphene tri sheets. Results Phys..

[cit44] Aarik L., Mändar H., Ritslaid P., Tarre A., Kozlova J., Aarik J. (2021). Low-Temperature Atomic Layer Deposition of α-Al_2_O_3_ Thin Films. Cryst. Growth Des..

[cit45] Chai Z., Liu Y., Li J., Lu Z., He D. (2014). Ultra-thin Al2O3 films grown by atomic layer deposition for corrosion protection of copper. RSC Adv..

[cit46] Mäkelä M., Timo Hatanpää T., Mizohata K., Meinander K., Niinistö J., Räisänen J., Ritala M., Leskelä M. (2017). Studies on Thermal Atomic Layer Deposition of Silver Thin Films. Chem. Mater..

[cit47] Gao Y., Walsh M., Liang X. (2020). Atomic layer deposited conformal ceramic coatings for anti-corrosion of Ag nanoparticles. Appl. Surf. Sci..

[cit48] Johnson R. W., Hultqvist A., Bent S. F. (2014). A brief review of atomic layer deposition: from fundamentals to applications. Mater. Today.

[cit49] Oviroh P. O., Akbarzadeh R., Pan D., Coetzee R. A. M., Tien-Chien Jen T.-C. (2019). New development of atomic layer deposition: processes, methods and applications. Sci. Technol. Adv. Mater..

[cit50] Smith J. B., Daniel Hagaman D., Ji H.-F. (2016). Growth of 2D black phosphorus film from chemical vapor deposition. Nanotechnology.

[cit51] Saleem M. R., Ali R., Khan M. B., Honkanen S., Turunen J. (2014). Impact of atomic layer deposition to nanophotonic structures and devices. Front. Mater..

[cit52] Zhang J., Li Y., Cao K. (2022). *et al.*, Advances in Atomic Layer Deposition. Nanomanuf. Metrol..

[cit53] Ansari G., Pal A., Srivastava A. K., Verma G. (2023). Detection of hemoglobin concentration in human blood samples using a zinc oxide nanowire and graphene layer heterostructure based refractive index biosensor. Opt. Laser Technol..

